# Clinically relevant antibiotic resistance genes are linked to a limited set of taxa within gut microbiome worldwide

**DOI:** 10.1038/s41467-023-42998-6

**Published:** 2023-11-14

**Authors:** Peter J. Diebold, Matthew W. Rhee, Qiaojuan Shi, Nguyen Vinh Trung, Fayaz Umrani, Sheraz Ahmed, Vandana Kulkarni, Prasad Deshpande, Mallika Alexander, Ngo Thi Hoa, Nicholas A. Christakis, Najeeha Talat Iqbal, Syed Asad Ali, Jyoti S. Mathad, Ilana L. Brito

**Affiliations:** 1 Meinig School of Biomedical Engineering, Cornell University, Ithaca, NY USA; 2 Oxford University Clinical Research Unit (OUCRU) in Ho Chi Minh City, Ho Chi Minh city, Viet Nam; 3 Aga Khan University, Karachi, Pakistan; 4 Johns Hopkins University Clinical Trials Unit, Byramjee Jeejeebhoy Government Medical College, Pune, Maharashtra India; 5 Centre for Tropical Medicine, Nuffield Department of Medicine, University of Oxford, Oxford, UK; 6 Microbiology Department and Center for Tropical Medicine Research, Ngoc Thach University of Medicine, Ho Chi Minh city, Vietnam; 7 Yale University, New Haven, CT USA; 8 Weill Cornell Medicine, New York, NY USA

**Keywords:** Bacterial genes, Antimicrobial resistance, Microbiome

## Abstract

The acquisition of antimicrobial resistance (AR) genes has rendered important pathogens nearly or fully unresponsive to antibiotics. It has been suggested that pathogens acquire AR traits from the gut microbiota, which collectively serve as a global reservoir for AR genes conferring resistance to all classes of antibiotics. However, only a subset of AR genes confers resistance to clinically relevant antibiotics, and, although these AR gene profiles are well-characterized for common pathogens, less is known about their taxonomic associations and transfer potential within diverse members of the gut microbiota. We examined a collection of 14,850 human metagenomes and 1666 environmental metagenomes from 33 countries, in addition to nearly 600,000 isolate genomes, to gain insight into the global prevalence and taxonomic range of clinically relevant AR genes. We find that several of the most concerning AR genes, such as those encoding the cephalosporinase *CTX-M* and carbapenemases *KPC*, *IMP*, *NDM*, and *VIM*, remain taxonomically restricted to Proteobacteria. Even *cfiA*, the most common carbapenemase gene within the human gut microbiome, remains tightly restricted to *Bacteroides*, despite being found on a mobilizable plasmid. We confirmed these findings in gut microbiome samples from India, Honduras, Pakistan, and Vietnam, using a high-sensitivity single-cell fusion PCR approach. Focusing on a set of genes encoding carbapenemases and cephalosporinases, thus far restricted to *Bacteroides* species, we find that few mutations are required for efficacy in a different phylum, raising the question of why these genes have not spread more widely. Overall, these data suggest that globally prevalent, clinically relevant AR genes have not yet established themselves across diverse commensal gut microbiota.

## Abstract (web summary)

Antimicrobial resistance genes (ARGs) in commensal gut bacteria may act as a reservoir for acquisition by pathogens. Here, the authors assess the distribution and transfer potential of ARGs in gut microbiomes and find that clinically important ARGs are taxonomically restricted despite being associated with mobile plasmids

## Introduction

Preventing the spread of multidrug- and pandrug-resistant pathogenic bacteria remains a primary focus of global health efforts^1^. Recognition that the commensal gut microbiota harbor extensive numbers of diverse AR genes^2–4^, engage in horizontal gene transfer (HGT) at higher rates than microbiota in other environments^2,5,6^, and may serve as a stable reservoir for pathogenic acquisition^5,7,8^ has prompted broader AR surveillance beyond clinical isolates^9–13^. The potential for the spread of AR genes between taxa through HGT^14–17^ has raised further concern, especially for *mcr* genes, which confer resistance against colistin, and carbapenemases. However, many of the annotated AR genes observed to transfer within microbiomes, including many common Class A beta-lactamase variants^18^, do not confer phenotypic resistance to clinically relevant antibiotics. Understanding the global spread and HGT potential of clinically relevant AR genes—particularly those associated with ‘last resort’ antibiotics—would be helpful, yet surprisingly little is known about the gene-taxa associations within microbiomes at large.

Here, using a combined analysis of 14,229 human gut metagenomes and nearly 600,000 isolate genomes, we find that certain AR genes are prevalent throughout global populations’ microbiomes; however, thus far associated with few taxa. To determine whether there may be any additional bacterial hosts for twelve such clinically relevant genes, we applied a single-cell fusion PCR method to associate the genes with the 16S rRNA sequence of the host. We find that these genes are harbored by the set of taxa in which they were previously observed, despite being mobilizable, and, in some instances, functional in taxonomically distant organisms. This study challenges the notion that AR genes have spread more broadly within the gut microbiome than currently realized.

## Results

### Many clinically relevant AR genes are found in a few isolated taxa

**Fig. 1:**
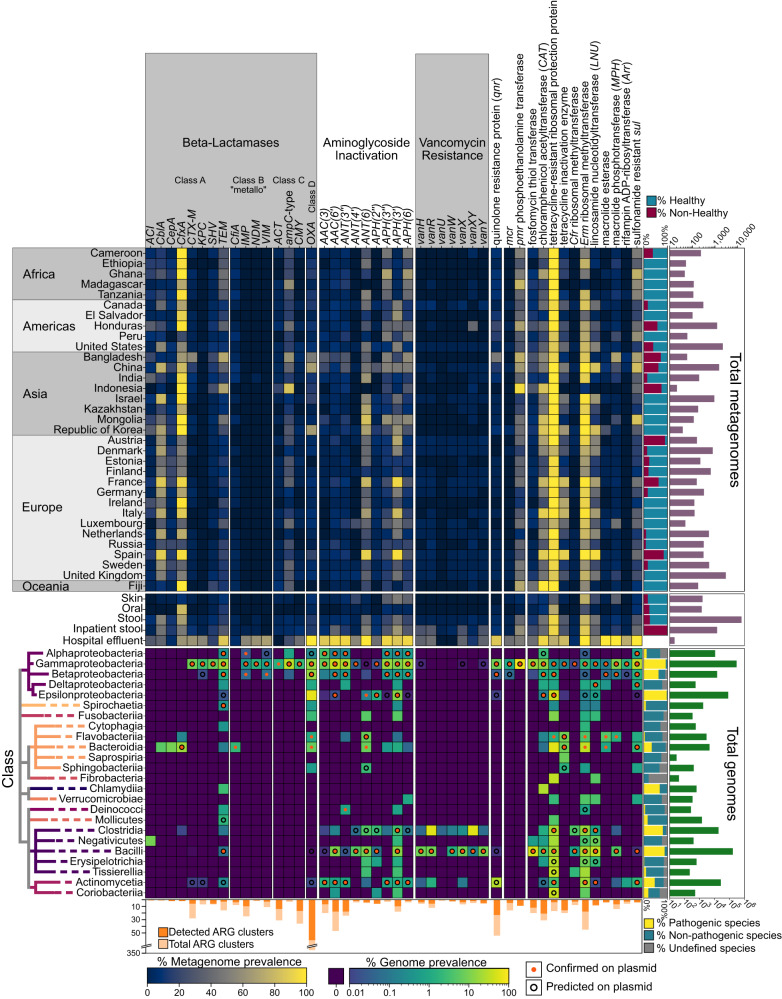
Distribution of clinically relevant AR genes across global human-associated microbiomes globally and cultured isolates. The top heatmap shows the prevalence of AR gene families in human gut-associated microbiomes sequenced from each country. Immediately below is a heatmap showing the total prevalence in gut, skin and oral microbiomes. The bottom heatmap shows the prevalence of AR gene families in sequenced isolates by taxonomic class. Color scales represent the percent of metagenomes (top) or isolates (bottom) for which the gene family was detected. At right of the top heatmap are two barplots, one showing the proportion of samples from heathy (blue) and not-healthy (red) individuals, as classified by curatedMetagenomicDataCuration^19^, and a second showing the total number of samples from each country. At right of the bottom heatmap are two barplots, the first showing the proportion of isolates from pathogenic (yellow), non-pathogenic (red), or unannotated species (gray), as determined by CARD, and the second showing the total number of isolates surveyed per class. At the bottom, a barplot shows the total number of AR gene varients in each gene family (light orange) and the number detected in this study (dark orange) in the metagenomic datasets. Nearly all genes in each family shown were identified in the isolate genomes.

To define the established taxonomic breadth of clinically relevant AR genes and their distribution within human-associated microbiota, we first examined the global prevalence of 46 known AR gene families in 14,229 human gut microbiome samples, as well as an additional 248 oral and 261 skin microbiome samples spanning 33 countries compiled from the curatedMetagenomicData package^19^ (Supplementary Data 1–3). We then coupled this with a taxonomic analysis of AR genes within nearly 600,000 sequenced genome isolates from GenBank (Supplementary Data 4, 5). The 46 clinically relevant gene families selected confer resistance to 18 classes of antibiotics, defined by the Comprehensive Antibiotic Resistance Database (CARD)^20^ (Supplementary Data 6), and largely overlap with the World Health Organization’s List of Essential Medicines^21^ (Fig. 1, all gene families shown in Supplemental Fig. 1). We examined the prevalence of all identifiable AR markers in CARD, excluding efflux pumps, as their role in antibiotic resistance versus other biological functions may be difficult to annotate and confirm^22^ and they are less frequently mobilized^23^. We further excluded metabolic genes recently shown to confer resistance^24^, due to the difficulties of distinguishing their physiologic role from their role in AR.

Notwithstanding small differences in read depth, AR gene detection method, or individuals’ health status (Supplemental Figs. 2–4), AR gene families exhibited a relatively even geographic distribution across continents but differed between the gut microbiomes of Western and non-Western populations, as defined by Pasolli et al.^25^ (Supplemental Fig. 5). This split has been observed in other potential AR gene reservoirs, such as sewage, and bacterial isolate sources and may be explained by socio-economic factors^26–29^. AR gene families also exhibited a bimodal distribution, such that they were either detected in many countries or very few (Supplemental Fig. 6). Gene-specific prevalence in the oral and skin microbiomes mirrored, albeit with reduced prevalence, those found within the gut (Fig. 1). Together, these findings concretely illustrate the reality that AR is a “global commons” problem.

We next assessed the host ranges of different AR gene families in isolate genomes. Here, certain taxa tend to harbor a disproportionately higher number of genes (Fig. 1). The Gammaproteobacteria, Betaproteobacteria, and Bacilli are among those taxa, though these organisms tend to be over-represented, as they comprise pathogenic strains, several representing prominent AR threats, and have thus been surveyed to a greater extent. Actinomycetia, which includes *Streptomyces*—known to be a source of AR genes—also harbor many AR gene families.

It may be expected that gene families with greater taxonomic range are generally more prevalent globally. That is the case (Supplemental Fig. 7, two-sided Pearson’s *ρ* = 0.558, *p*-value = 0.000896), however, unexpectedly, a subset of AR gene families was found to be restricted to a single taxonomic class (e.g., NDM and *CMY*) (Fig. 1, Supplemental Fig. 1). This restricted distribution was particularly surprising given their association with plasmids^30,31^, which we confirmed with our analysis of AR gene-plasmid associations (Fig. 1). Two cephalosporinase genes *cepA* and *cblA*, as well as the carbapenemase gene *cfiA*, were similarly found to be confined to the genus *Bacteroides*, despite this being the most abundant and prevalent genus in Western countries^32^, theoretically providing ample opportunity to transfer.

### Factors related to AR gene prevalence and taxonomic host range

We next sought to determine the selective pressure imposed by antibiotic use on global AR geographic distribution and taxonomic spread. Antibiotic use on an individual level is generally associated with an increase in AR gene abundance in gut microbiomes^11,33,34^. Historical data on global antibiotic use however are sparse, due to the lack of centralized reporting that accounts for human and animal consumption, and environmental use. Nevertheless, recent data from the World Health Organization (WHO) representing 65 countries^35^ afford the opportunity to examine global use of oral and parenteral use of antibiotics with AR gene prevalence in human gut microbiomes.

We expected higher global antibiotic use to correlate with greater AR gene prevalence in microbiomes. Yet, this was not the case. Beta-lactams, including penicillins, categorized as Access drugs, under the WHO’s suggested use categories of Access, Watch and Reserve, were the single most consumed category of antibiotics worldwide (36.7% ± 12.25 of total consumption per country)^35^. Despite the copious use of broad-spectrum beta-lactams, only two beta-lactamase genes, *cfxA* and *cblA*, were found to be highly prevalent in human gut microbiomes (Fig. 1). This was unexpected, as beta-lactamases are diverse, and many, even those that are most concerning, confer resistance to multiple beta-lactams, including amoxicillin, one of the most widely consumed drugs^35^.

The most striking observation was a general lack of carbapenemases in gut microbiomes. Watch-group beta-lactams, including carbapenems, penems, monobactams, and third-, fourth-, and fifth-generation cephalosporins, comprised the second most consumed category of antibiotics (13.99% ±8.05 of total use per country). Aside from *cfiA*, which has surprisingly high prevalence, we identified only 8 individuals out of 14,229 harboring any other carbapenemase in their gut microbiomes (Supplementary Data 2, 3). This finding contrasts with reports of nosocomial spread of these genes^36,37^. For example, another carbapenemase, *NDM-1*, was first identified in a clinical *Klebsiella pneumoniae* isolate from an inpatient who was simultaneously colonized with commensal *Escherichia coli* carrying the gene^38^. Despite this potential reservoir, the gene family *NDM* was observed in only 3 samples worldwide. Similarly, *OXA-48*, the only gene in the *OXA* family with significant carbapenemase activity^39,40^ (which, unlike other phylogeny-based AR gene families, is based on phenotypic resistance to oxacillin alone), was identified in only 5 metagenomes. By contrast, chloramphenicol-resistance genes were found to be pervasive across global metagenomes, even though chloramphenicol has long been considered a last-resort antibiotic due to toxicity and has relatively low usage (0.29% ± 0.68 of total use per country)^35^.

Since the majority of the metagenomic samples we analyzed were from outpatient cohorts (Supplemental Fig. 4), we wanted to directly compare the AR gene profiles in these samples with those in other suspected reservoirs, including gut metagenomes obtained from inpatient cohorts, hospital effluent, wastewater treatment plants, and livestock^41–55^ (Supplementary Data 7–9). Hospital effluent is the only suspected reservoir that was strongly enriched for clinically relevant AR genes, including *mcr* and *CTX-M*, and the carbapenemase-encoding genes *KPC*, *NDM*, *VIM*, and *IMP* (Fig. 1, Supplemental Fig. 1). We hypothesize that the enrichment of carbapenemases in hospital effluent is due to aerobic conditions selecting for Proteobacteria, which commonly harbor diverse AR genes.

**Fig. 2:**
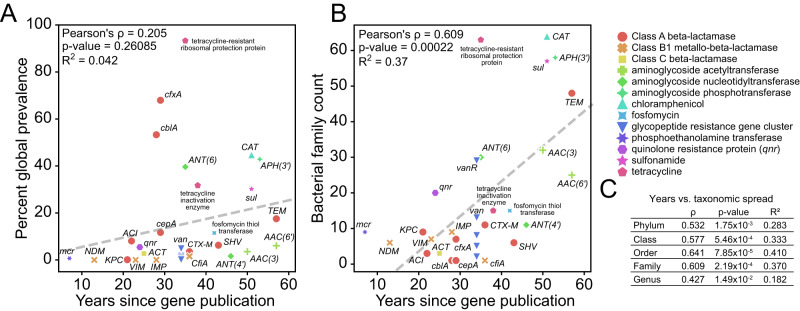
Time allows for genes to become more prevalent and spread more broadly. **A**, **B** For each gene family, the time since initially reported in the scientific literature, a proxy for AR gene emergence, is plotted against the prevalence in human gut microbiomes (left) and number of genera in which the gene is found (right). Marker color/shape indicate the AR gene class. Two-sided Pearson’s *ρ*, *p*-value and *R*^2^ are reported. **C** Statistics (two-sided Pearson’s *ρ*, *p*-value and *R*^2^) are reported for the years since AR gene emergence and taxonomic spread computed at different taxonomic levels.

To address the possibility that AR gene spread, both geographically and between taxa, may simply be the result of time, we used the first report of an identified AR gene family in the literature, a previously established metric^56^, as a proxy for the year at which it emerged (Supplementary Data 6). Whereas time since emergence has had little bearing on the global prevalence of AR gene families (Fig. 2A), time has allowed for greater taxonomic distribution (two-sided Pearson’s *ρ* = 0.609, *p*-value = 2.2 ×10^−4^, at the family-level) (Fig. 2B, C). Genes within the *mcr* family serve as a counterexample, having emerged relatively recently^57^ and, despite their low prevalence in the human gut microbiome, have rapidly spread to many taxa (21 genera across 2 classes). Although polymyxins, to which *mcr* genes confer resistance, are infrequently prescribed today, they have historically been used for growth promotion in livestock. Their prevalence may have been limited by the disproportionate fitness defects caused by these genes compared to other AR genes^58^. Rather, their persistence within communities is thought to be stabilized by being situated within transposons on conjugative plasmids that may be maintained by other selective means^59,60^.

### Lack of hidden taxonomic reservoir of clinically relevant AR genes in gut microbiomes

Because the taxonomic diversity of the AR gene reservoir within human gut microbiomes is unknown, we tested whether clinically relevant AR gene family-taxa associations could be explained solely by using a collection of isolates sequenced thus far. To this end, we examined all 14,229 gut metagenomes from curatedMetagenomicData for the presence of at least one known host for each AR gene family. Save for a few AR gene families in a small number of people, isolate-based associations alone were sufficient to explain the distribution of AR gene families (Supplemental Fig. 8), hinting at the lack of hidden AR gene reservoirs. Only *mcr* genes in 6 total individuals lacked previously identified hosts at the phylum level, indicating that either there is an undefined reservoir for *mcr* genes, or known hosts have too low abundances to detect.

To experimentally elucidate AR gene-taxa associations within gut microbiomes, we applied OIL-PCR (one-step isolation and lysis PCR), a sensitive, culture-independent single-cell fusion PCR approach to associate extrachromosomal DNA with their associated genomes^61^. OIL-PCR overcomes technical limitations which have, until now, obscured our ability to assay the full taxonomic range of these genes. Metagenomic genome assemblies are neither able to robustly associate plasmids nor integrated mobile elements with specific bacterial hosts. And while culture-based screening can provide a window into AR host range, surveying the entire gut microbiota across many samples may be impractical due to diverse culturing conditions^62–64^.

**Fig. 3:**
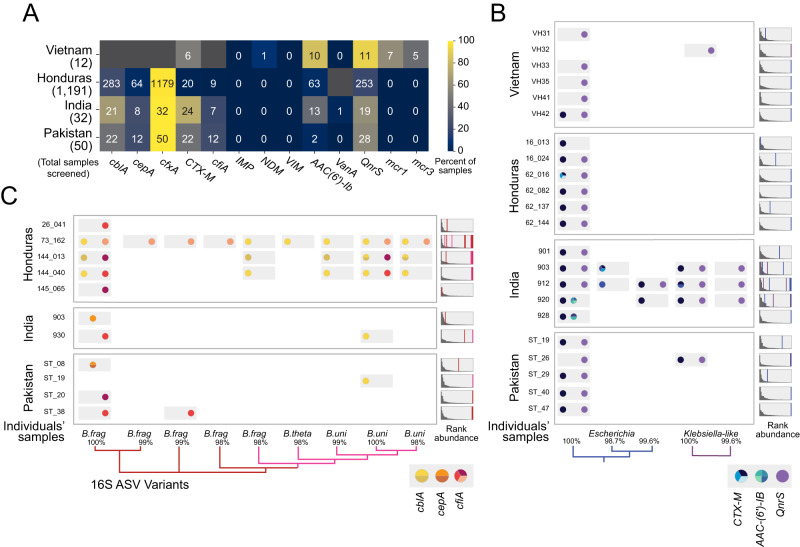
OIL-PCR confirms that select AR genes are taxonomically restricted. **A** A heatmap showing the number and percentage of gut microbiome samples that were positively screened for each AR gene, by quantitative PCR (for Vietnam, India and Pakistan) or metagenomic read alignment (Honduras). Bolded AR genes were further screened for taxonomic associations by OIL-PCR. Dark gray boxes indicate qPCR reactions that were not performed. **B** OIL-PCR was performed to detect taxonomic associations for *CTX-M*, *AAC-(6’)-IB*, and *qnrS* in positive gut microbiome samples from the international cohorts. Each row represents an individual’s gut microbiome sample. Colors represent AR gene variants. Phylogenies show the relationships between detected ASVs. The percent similarity is listed. To the right of each sample is a rank-ordered distribution of abundances of the individual ASVs in the individual’s gut microbiome, as determined by 16 S rRNA sequencing. Lines, colored according to the species designated in the phylogenies, are placed at the rank of the ASVs detected by OIL-PCR in each sample. **C** OIL-PCR results performed in *cfiA*, *cblA*, and *cepA*, displayed in the same manner as in (**B**). 31 individuals’ samples are shown in (**B**) and (**C**), with two individuals having been tested for the genes in both (**B**) and (**C**).

To identify candidate samples for OIL-PCR, we tested 100 gut microbiome samples from India, Pakistan, and Vietnam for the presence of twelve clinically relevant AR genes of interest (*cblA*, *cepA*, *cfxA*, *CTX-M*, *cfiA*, *IMP*, *NDM*, *VIM*, *AAC(6’)-Ib*, *VanA*, *QnrS*, *mcr1*, and *mcr3)* using a probe-based quantitative PCR approach (Fig. 3A, Supplementary Data 10). We also computationally screened 1187 additional samples from Honduras, for which we had metagenomes, by aligning reads to AR genes (same as in Fig. 1). These results validated the prevalence trends observed in our analysis of global gut microbiomes. We selected thirty-one samples for OIL-PCR and focused on six genes: *CTX-M*, *aac(6’)-Ib-cr* and *qnrS*, which have been shown to be mobilizable, and *Bacteroides*-specific *cblA*, *cepA* and *cfiA* (Fig. 3B, C, Supplementary Data 10, 11). We found that all six genes were restricted to the genera expected based on the isolate genomes and included the presence of multiple amplicon sequence variants (ASVs) within the same sample, each carrying a distinct set of AR gene variants, with relative abundances as low as 0.0044%. We identified several novel associations: *cfiA* with *B. uniformis* and *cblA* with *Bacteroides thetaotaomicron* and *B. uniformis*, though we were unable to exclude the possibility that these associations resulted from chimeras between highly similar 16S rRNA sequences formed during library preparation. These results thus provide orthogonal experimental support for the absence of a prevalent unculturable commensal reservoir for these genes.

### *Bacteroides*-specific beta-lactamases can be transferred and are functional in Enterobacteriaceae

Commensal beta-lactamases conferring resistance against clinically relevant antibiotics, including *cepA*, *cblA*, *cfxA* and *cfiA*, have the potential to exacerbate AR infections if transferred to pathogens within the Gammaproteobacteria. Aside from *cfxA*, which has been sporadically found in other phyla, these genes are currently restricted to the *Bacteroides*. While inter-phylum HGT is considered a rare event—with few transfers recorded between isolates within the Bacteroidetes and Gammaproteobacteria^5,65^—the high relative abundance of Bacteroidetes within gut microbiomes might be expected to result in higher contact rates with enteric pathogens, and therefore an increased probability of HGT. We therefore sought to explore potential barriers of beta-lactamase HGT beyond *Bacteroides*.

**Fig. 4:**
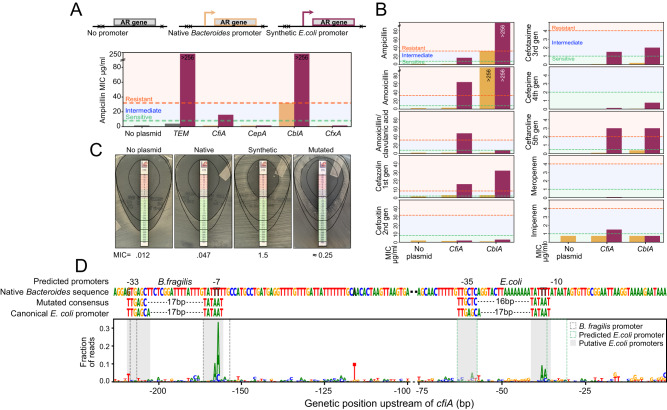
*Bacteroides*-specific beta-lactamases CfiA and CblA are functional in *E. coli*. **A** AR genes TEM, *cfiA*, *cepA*, *cblA*, and *cfxA* were cloned onto plasmids either with no promoter (TEM only), their native *Bacteroides* promoter, or a synthetic *E. coli* promoter, transformed into *E. coli* and tested on ampicillin to determine their minimum inhibitory concentrations (MIC). **B** MICs to various antibiotics were determined using antibiotic strips on agar for *E. coli* carrying *cfiA* and *cblA*, placed under its native *Bacteroides* promoter (yellow) and an *E. coli-*specific promoter (purple). *E. coli* without plasmid were used as a control. **C** *E. coli* tested for growth on agar plates treated with cefotaxime strips. *E. coli* harboring no plasmid, or a plasmid harboring *cfiA* under control of its native *B. fragilis* promoter, the synthetic *E. coli* promoter, or a mutant library of *B. fragilis cfiA* promoters (Mutated). **D** Histogram of mutations within the *B. fragilis* native promoter identified in the mutated *cfiA* promoter library after 18 h of growth in 0.5 ug/ml of cefotaxime. The native *B. fragilis* promoter sequence is shown, as are the predicted *B. fragilis* and *E. coli* promoters in the native sequence, the mutated consensus sequence and a depiction of the canonical *E. coli* promoter.

We first investigated if these AR genes could confer a resistance phenotype in *E. coli*. We placed *cepA*, *cblA*, *cfxA*, and *cfiA* under control of either their native promoter or a synthetically designed promoter active in *E. coli*^66,67^. We transformed each construct into the auxotrophic strain DH5α, for the purpose of biocontainment, and measured the minimum inhibitory concentration (MIC) of each AR gene to ampicillin (Fig. 4A). Both *cblA* and *cfiA* are shown to be functional when expressed by an *E. coli* promoter, yet only *cblA* was also functional under control of its native *Bacteroides* promoter, suggesting that *cblA* could provide immediate selective benefit in the presence of ampicillin. We further found that when adequately expressed, *cblA* and *cfiA* were capable of conferring *E. coli* with resistance to other next-generation cephalosporins (Fig. 4B). *cfiA* also provides resistance to the beta-lactamase inhibitor clavulanic acid and the carbapenem imipenem, largely matching the literature^68–70^. Furthermore, the expression of these genes in *E. coli* did not result in a significant growth defect, when compared to a wildtype strain or a strain expressing a *TEM* beta-lactamase commonly found in *E. coli* (Supplemental Fig. 9).

We were especially intrigued by *cfiA* because of its clinical relevance and activity in *E. coli*. We investigated the evidence for mobilization of *cfiA*. The mobilizable plasmid on which *cfiA* is situated, pBFUK1, has been shown to transfer from *B. uniformis* to *E. coli*, and vice versa^71–73^. Furthermore, *cfiA* is carried on a composite transposon composed of two IS1380-like elements. While the transposase and inverted repeat sequences could be found in Gammaproteobacteria, a complete transposable element containing both repeats was only found in *Bacteroides*.

We hypothesized that some minor mutations in the native *B. fragilis cfiA* promoter would allow its expression in *E. coli*, especially considering the presence of a predicted *E. coli* promoter downstream of the native *B. fragilis* transcriptional start site. We screened a library of randomly mutated *B. fragilis cfiA* promoters for increased MIC to cefotaxime, a third-generation cephalosporin, in *E. coli* (Fig. 4C), finding that most mutations occurred either within the *B. fragilis* promoter binding site or within the predicted *E. coli* promoter, thus creating a TATAAT box within proper range of the −35 region with only two mutations (Fig. 4D). This suggests that neither expression, function, nor transfer are the factors limiting the taxonomic spread of the carbapenemase *cfiA*.

## Discussion

Surveillance of AR genes within the human microbiome is grounded on the perceived threat that these genes may be transferred to pathogens. Given the prevalence of AR genes in the gut, the selective pressures imposed by antibiotics, and the observation that the gut environment is conducive to HGT^5,17,62,74–78^, it is expected that AR genes would have been transferred to a wide range of organisms, thereby creating a diverse reservoir of AR genes. While this is the case for some AR genes, others have surprisingly restricted host associations. This is most striking for commensal-associated beta-lactamases, despite their high global prevalence.

While antibiotic stewardship has emerged as a critical strategy to reduce the circulation of multi-drug resistant pathogens, it may be less influential in shaping the global reservoir of AR strains within the human gut. For example, *Bacteroides* often carry aminoglycoside-resistance genes from Proteobacteria, despite their intrinsic resistance to this drug^79^, essentially decoupling antibiotic-mediated selection from use, as also shown by others^80^. This dissociation may be due to the functions of co-localizing genes on AR gene-encoding plasmids^81^, alternative physiologic functions of AR genes^24^, multi-drug efflux pumps that may provide sufficient resistance, and differences in antibiotic concentrations felt across the intestinal microenvironment^82^, among other reasons^83^. Microbial community effects may also play a major role. For instance, beta-lactamases are secreted^84^, thereby mitigating the effects of antibiotics on susceptible bacteria within the community. Bacteria are similarly protected while situated within a biofilm^85,86^. Changes to community structure also have the potential to affect the landscape of HGT by modulating contact rates between organisms^87^. These examples illustrate some of the ways in which selective pressures imposed by antibiotics may not directly affect sensitive bacteria or bacteria carrying resistance markers.

Although there appears to be limited societal transmission of certain AR strains, nosocomial transmission of these strains may be markedly higher. This, along with increased antibiotic use, may promote the emergence of novel AR strains, especially within the hospital environment. The strong association of time with the taxonomic distribution of AR genes suggests that their emergence and spread within and across gut microbiomes may reflect varying rates of engraftment and persistence^34,88,89^. Curtailing the expanding reservoir of AR strains may therefore require strategies tailored to specific AR gene families. Indeed, fecal microbiota transplantation has been shown to effectively reduce the carriage of vancomycin-resistant *Enterococci*^90^. Phage and gene editing machinery have similarly been shown to reduce the carriage of AR genes^91–93^. Challenges notwithstanding, the geographic and taxonomic spread of AR genes within human microbiomes has grown with time, highlighting the need for continued surveillance of isolates and microbiomes and more explicit studies on the factors that allow, or restrict, certain AR genes from establishing a global reservoir within commensal gut microbiomes.

## Methods

### Public metagenome acquisition

The curatedMetagenomicData datasets package^25^ was used to compile a metadata table for all metagenomes included in the Waldron dataset. (Supplementary Data 1). Samples were filtered for at least 5 million reads. Fastq files were downloaded from EBI using NCBI accession numbers acquired from the metadata to query FTP links (wget -qO-“https://www.ebi.ac.uk/ena/portal/api/filereport?accession = <NCBIaccession > &result=read_run&fields=fastq_ftp”). Raw reads were then downloaded using the compiled FTP links (wget -c --retry-connrefused -P <download_location > <ftp_link > ). Due to the large variability in Illumina sequencing methods across datasets, all metagenomes were processed as single-end reads by concatenating the fastq read files into a single file. For analysis of inpatient, waste-water, air, and animal metagenomes, metadata and accession numbers were manually curated from multiple studies and downloaded with the same method (Supplementary Data 7).

### AR gene profiling of metagenomes

Concatinated fastq files were rimmed with bbTools version 38.96 (*bbduk.sh in=infile.fastq out=trimmed.fastq ref=adapters ktrim* = *r k* = *23 mink* = *11 hdist* = *1 tpe tpo*), and aligned to the Comprehensive Antibiotic Resistance Database^20^ (CARD) protein homolog model database using KMA^94^ version 1.4.3 (*kma -i trimmed.fastq -o kma.txt -t_db card_db*). KMA results were analyzed using a custom python script (kma_result_analysis_V8.ipynb). In short, positive hits were strictly filtered for template identity over 90%, template coverage over 80%, and p-values under 0.0001. Many hits within the KMA results mapped to single nucleotide gene variants within the CARD database, so CD-HIT^95^ was used to cluster the database to 99% identity to reduce redundancy in the dataset. KMA results were then combined based on the CARD clustering. AR gene family profiles were assembled by counting the presence or absence of AR gene families within each metagenome. Individual sample profiles were then used to calculate the percent prevalence of each gene family by country and in all samples grouped by body site.

To confirm our results, we conducted an alternative analysis using HUMAnN3 data acquired from the curatedMetagenomiData package. In short, we ran the Resistance Gene Identifier (RGI)^20^ version 5.2.0 on the UniRef90 database used by HUMAnN3 to identify resistance genes. We used the RGI results to build resistance gene profiles for each metagenome by filtering the HUMAnN3 output tables. These profiles were then processed similarly to the KMA analysis to build a heatmap for gene prevalence by country.

### Acquisition of isolate genome and plasmid sequences

Metadata for all genomes withing GenBank were acquired through NCBI (https://ftp.ncbi.nlm.nih.gov/genomes/genbank/assembly_summary_genbank.txt 2022-2-11). Only Bacterial genomes with an assigned genus were used. For genomes marked as “excluded from RefSeq”, only those assonated as “from large multi-isolate projects” and “fragmented assemblies” were used, excluding those derived from environmental sources, metagenomes, or single-cell sequencing. *Salmonella enterica* and *Escherichia coli* were both randomly subsampled to 100 thousand genomes each due to their overrepresentation in the dataset. FTP links were then compiled from the metadata and sequences downloaded with wget. Plasmid sequences were similarly acquired from RefSeq’s plasmid database (https://ftp.ncbi.nlm.nih.gov/genomes/refseq/plasmid/).

### AR gene profiling of isolates

RGI^20^ version 5.2.0 and the CARD database version 3.1.4 was used to detect AR genes in genomes and plasmids (*rgi main --input_sequence inGenome.fasta --output_file rigout.txt --input_type contig --local –clean --alignment_tool DIAMOND --num_threads 5 --split_prodigal_jobs --include_loose*). Using a custom python script (*process_rgi_V14.ipynb*), RGI hits were filtered to remove “loose” hits and AR gene family profiles were constructed for each genome similar to the metagenomic profiles (Supplementary Data 5). AR gene family prevalence was then calculated for each Class with at least 10 representatives. Gene family/taxa associations with <2 detections were removed. Isolate contigs containing AR genes were classified as genomic or plasmid using PlasForest^96^ version 1.4 (*python3 PlasForest.py -f -r --threads 4 -i AR_Contigs.fasta -o out_plasforest.csv*)

### Human participant consent and sample acquisition

Human participant research was approved by the following committees: Cornell University Institutional Review Board (#1706007261, # 1702006922, # 1609006586), Aga Khan University Ethics Review Committee (#2019-0550-5166), Ethics Committee of the University of Oxford (OxTREC 38-15) and of Tien Giang Hospital Institutional Review Board (278/BVĐK), the Yale University Institutional Review Board (#2000020688), the Indian BJ Government Medical College Ethics Board, and Weill Cornell Medicine Institutional Review Board (#1503016041, #1504016114). Honduran study participants from 9 isolated villages in the western highlands of Honduras that were part of larger population-based cohort assembled for a different purpose^97^ were asked to take part in this study. The goal of the microbiome sampling was to be as comprehensive as possible. Pakistani study participants comprised adults (over the age of 18) recruited via the existing community-based antimicrobial surveillance system established by the two union councils of the Matiari district. Participants were stratified based on ethnicity/caste and tribe and random representatives were chose across the communities. Vietnamese participants comprised adult farmers (over the age of 18) involved in studies conducted by the Oxford University Clinical Research Unit (OUCRU) in Vietnam. Indian participants comprised a subset of pregnant women enrolled in the PRACHITi study in Pune, India^98^. All women were over the age of 18 who presented to the antenatal clinic at BJ Government Medical College in Pune, India, with gestational age between 13–34 weeks. After obtaining consent, all human research participants from Honduras, India, Pakistan, and Vietnam were provided with stool acquisition kits and instructed on how to self-collect the samples, store and transport samples to the study coordinators. After voiding, participants kept samples cold using cold packs until they could be frozen at −80 °C.

### Sample processing, DNA extraction, and metagenomic sequencing

Aliquots of frozen stool samples from Honduras were shipped on dry ice to Cornell University. 200 mg of stool material was placed in 2 ml PowerBead Tubes with 2.8 mm ceramic beads (Qiagen, Cat. No.:13114-50) for DNA extraction using the Chemagic Stool gDNA Extraction Kit (Perkin Elmer, CMG-1076) on the Chemagic 360 Instrument (Perkin Elmer). Resulting genomic DNA quantity, quality, and purity were assessed via determination of the 260/280 nm for values of 1.7–2.0, and 260/230 absorbance ratios for values ≥ and 1% agarose gel electrophoresis to ensure that the gDNA was neither degraded nor displayed RNA contamination. Metagenomic libraries were prepared using KAPA Hyper Library Preparation Kit (KAPA Biosystems, Part#KK8504). Resulting libraries were sequenced on the Illumina NovaSeq (2×150).

### qPCR screening of samples

Gene variant sequences for each target gene were compiled from CARD and aligned in SnapGene. Primers targeting conserved regions were designed with 40–60% GC content and an annealing temperature of ~58 °C. Multiple primer sets were designed for each gene and tested for optimal amplification using the OIL-PCR^61^ master mix modified by omitting lysozyme and using a lower concentration of Phusion polymerase (1U per 50 µl of reaction). Isolates acquired from the CDC & FDA Antimicrobial Resistance Isolate Bank were used as templates for testing primers. Amplification quality was evaluated based on clean bands on a gel as well as efficient nested PCR amplification using NEB Luna Universal qPCR Master Mix. Primer/probe sets were designed based on the validated OIL-PCR primers, using the round 1 and fusion primers for amplification, and the nested round 2 primer sequence for the probes (Supplementary Data 10). Primers were tested against purified genomic DNA as a positive control, and purified metagenomic DNA as a negative. Lastly, primers and probes were tested in triplex combinations to check for cross-reactivity between sets. Metagenomic DNA was purified using Qiagen DNeasy PowerSoil Kit (12888-100). Reactions were multiplexed as shown in Supplementary Data 10 and run in duplicate 15 µl reactions with 3 µl of genomic DNA on the Quant Studio 7 Pro for 50 cycles. Positive detections were defined by detected amplification across duplicates.

### 16S rRNA library preparations

16S rRNA libraries were prepared in duplicate 20 µl reactions as follows: 20 U/ml of Phusion Hot Start Flex DNA polymerase, 1x HF Buffer, 2 μM dNTPs, 200 nM of i519F and i786R universal 16 S rRNA Illumina primers, and 25 ng of purified DNA template. Reactions were amplified as follows: 95 °C for 3 min, followed by 22 cycles of 95 °C for 5 s, 52 °C for 30 s, and 72 °C for 30 s, before final extension of 72 °C for 5 minutes. Duplicate reactions were pooled and treated with heat-labile Exonuclease I (NEB M0293L) to degrade the primers (10U ExoI per reaction, incubated at 37 °C for 25 min followed by 5 min at 80 °C). ExoI treated 16S amplicons were indexed as described previously^61^.

### OIL-PCR

Nycodenz purification of cells and OIL-PCR was performed with automation as described previously with some adjustments^61^. To remove clumped cells that can signal false gene associations, purified cells were passed through a 5 µm nylon syringe filter. To improve the sensitivity of OIL-PCR, we increased the input of cells 10-fold to 400 thousand cells per reaction based on previous experiments showing that OIL-PCR is more accurate with low abundance cells^61^. In addition, after performing the nested PCR step, multiplexed reactions were recombined and indexed in a single PCR reaction. Lastly, instead of performing Ampure bead purification between the nested PCR and indexing PCR, we used ExoI treatment to degrade nested primers as described for the 16S rRNA library preparations.

OIL-PCR sequences were analyzed with a modified pipeline to sort pooled libraries and allow an analysis of individual ASV variants. Using the script “s1.1_run_mrg_trim_flt.qsub.sh” reads were first merged using usearch mergepairs version 11.0.667 (*usearch -fastq_mergepairs forward.fq -reverse reverse.fq -fastq_pctid 80 -fastq_minmergelen 350 -fastq_maxmergelen 470 -fastq_maxdiffs 10 -fastqout merged.fq*), and cutadapt version 3.4 was then used to trim primers (*cutadapt -a ^TARGET1_primer…16* *S_rev$ -a ^ TARGET2_primer… 16S_rev$ -a ^TARGET3_primer… 16S_rev$ --discard-untrimmed -o trimmed.fq merged.fq --cores* = *2*), before quality filtering with usearch (*usearch -fastq_filter trimmed.fq -fastq_maxee 0.5 -fastaout filtered.fa*).

Next the script “s2_combine_split_sort_V2.sh” was used to split target sequences from the 16S sequence using cutadapt to recognize the gene-specific fusion primer in each read (*cutadapt -a $target_fusion_primer --discard-untrimmed -o output_target.fa filtere.fa --suffix _target --cores* = *10*). Cutadapt was run separately for each fusion primer in multiplexed sequencing to simultaneously split and sort the target reads. Cutadapt was run a final time using the 519F 16S sequence to isolate the 16S portion of each read. Duplicate sequences were counted using usearch -fastx_uniques (*usearch -fastx_uniques output_target.fa -fastaout unique.fa -relabel target_uniq_ -sizeout*). Lastly, 16 S sequences were clustered into OTUs using usearch -cluster_otus (*usearch -cluster_otus uniques.fa -otus otus_out.txt -relabel otu -uparseout parsed_file.txt -minsize 1*) and taxonomy assigned using MOTHUR RDP classifier with the SILVA version 132 using the script “s3_cluster_assign_tax.sh”.

A custom python script (*s4_Tax_Blast_Connect_SNPS_V7.ipynb*) was used to compile taxonomic-gene associations while keeping track of ASV variants. In short, fasta files were read into tables and sequences were used as identifiers to merge information about the identity of the target gene, assigned taxonomy for the 16 S read, and count the number of reads for the association. A second python script was used to graph the output (*graphing_results_V4.ipynb*). In summary: 16 S sequencing results were imported and merged with the OIL-PCR association to provide community abundance of each ASV and OUT. Target reads were clustered to 99% identity with CD-HIT. Detected associations were filtered for detection across replicates.

### Testing activity of *Bacteroides* genes in *E. coli*

We built a strongly insulated expression vector for testing commensal AR genes to reduce the possibility of leaky gene expression from stray RNA polymerase. We designed a gene block containing two strong terminators upstream of TEM-1—a common ampicillin resistance gene used routinely for cloning—expressed from a synthetically designed promoter and RBS^66,67,99^. Plasmid 353 (pARG-test-insulated) was assembled with a p15A origin and chloramphenicol resistance gene amplified from plasmid pdCas9-bacteria (Addgene 269588, primers BH01/BH02) using Gibson assembly (NEBuilder Hi-Fi Assembly Master Mix).

Sequences for *cfiA-2*, *cfxA-2*, *cepA*, and *cblA-1* were selected from CARD, favoring those that were found on plasmids or other mobile elements. *cfiA* was selected from the pBFUK1 plasmid. Each gene was synthesized along with 250–350 bp upstream to capture the native promoter region. For the native promoter, the expression plasmid 353 was linearized with primers BH01/BH03 and the *ach* gene block was inserted using Gibson assembly. For the synthetic promoter constructs, the plasmid was linearized with primers BH01/BH04 and each gene block was amplified with primer BH08 paired with BH09-BH12 to remove the promoter. The fragments were then assembled with Gibson. For the promoter-less construct, plasmid 353 was amplified with primers BH03/BH05, phosphorylated, and circularized with T4 ligase.

- *cfiA2* from plasmid pBFUK1 (accession AB646744)
- *cfxA2* from *B. fragilis* strain DCMOUH0085B (accession CP037440)
- *cepA* from *B. fragilis* strain 74985 CTn86 mobile element (accession MW169430)
- *cblA1* from Uncultured *Bacteroides* sp. Clone AMP (accession MH883562)

### Antibiotic resistance MIC determination

Plasmid glycerol stocks were inoculated into 5 ml LB + chloramphenicol and grown at 37 °C overnight. Cultures were then diluted 1:10 in fresh LB and evenly spread onto Muller-Hinton agar (Hardy Diagnostics G45) with a cotton swab and allowed to dry. MIC test strips (Liofilchem) were then placed onto the agar with tweezers and plates were placed at 37 °C for at least 24 h before imaging. MIC measurements were evaluated following the manufacturer’s instructions.

### Random mutagenesis library generation and screening

The backbone of plasmid 364 (pARG_cfiA_native) was amplified with primers BH03/BH97. The promoter region was amplified with primers BH98/BH99 using the GeneMorph II random mutagenesis kit (Agilent 200550) with 300 ng template and 30 cycles. Mutated promoters were assembled into the backbone using Gibson assembly, diluted, and plated for individual colonies (~24 thousand). Colonies were pooled and stocks were frozen in 20% glycerol. Mutant libraries were grown overnight in 5 ml LB + chloramphenicol and diluted 1:100 in fresh LB + chloramphenicol with 0, 0.125, 0.5, 2, or 8 µg/ml of cefotaxime and allowed to grow overnight. Growth occurred at all antibiotic concentrations, likely due to compensatory genomic mutations. This was not observed for DH5a carrying *TEM*. Cultures were diluted 1:10 in TE and incubated 10 min at 99 °C to lyse. The raw lysate was used as a template for PCR with primers BI70/BI71 and processed following the 16 S rRNA library preparation protocol (ExoI treatment, index PCR, ampure cleanup, and pooling). Illumina reads were processed similar to 16 S rRNA reads, merging with usearch, trimming with cutadapt, quality filtering with usearch^100^, and counting uniques with usearch. Next, sequences were aligned to the original promoter with bwa mem version 0.7.17, converted to bam, sorted, and indexed with samtools^101^ version 1.15.1. The program bam-readcount^100, 102^ was used to count the bases at each position of the reads. A custom python script (s5_parse_tsv_and_graph_V05.ipynb) was then used to parse the bam-readcount output and the results were plotted using Logomaker^103^.

### Reporting summary

Further information on research design is available in the Nature Portfolio Reporting Summary linked to this article.

### Supplementary information

Supplementary Information

Peer Review File

Descirption of Additional Suppelmentary Data

Supplementary Data 1

Supplementary Data 2

Supplementary Data 3

Supplementary Data 4

Supplementary Data 5

Supplementary Data 6

Supplementary Data 7

Supplementary Data 8

Supplementary Data 9

Supplementary Data 10

Supplementary Data 11

Reporting Summary

## Data Availability

Sequence data was uploaded to the NCBI’s Short Read Archive (Honduras metagenomes: PRJNA999635; Neutropenic metagenomes: PRJNA999651; OIL-PCR and 16 S sequencing: PRJNA1001934).

## Code availability

Custom code is uploaded https://github.com/britolab/Global-and-taxonomic-ARG-prevelence10.5281/zenodo.10031964
