# Supplementary material for: Clinically relevant antibiotic resistance genes are linked to a limited set of taxa within gut microbiome worldwide
Source: Nat Commun. 2023 Nov 14;14:7366. doi: 10.1038/s41467-023-42998-6 (PMC10645880; doi:10.1038/s41467-023-42998-6)
Supplement: Supplementary file 1 — Supplementary Information [file 41467_2023_42998_MOESM1_ESM.pdf]

Supplementary Information

Clinically relevant antibiotic resistance genes are linked to a limited set of taxa within gut microbiome worldwide

Peter J. Diebold[1], Matthew Rhee[1], Qiaojuan Shi[1], Nguyen Vinh Trung[2], Fayaz Umrani[3], Sheraz Ahmed[3], Vandana Kulkarni[4], Prasad Deshpande[4], Mallika Alexander[4], Ngo Thi Hoa[2,5,6], Nicholas Christakis[7], Najeeha Iqbal[3], Asad Ali[3], Jyoti Mathad[8], Ilana Lauren Brito[1]

[1] Meinig School of Biomedical Engineering, Cornell University, Ithaca, NY, USA.
[2] Oxford University Clinical Research Unit (OUCRU) in Ho Chi Minh City, Viet Nam.
[3] Aga Khan University, Karachi, Pakistan.
[4] Johns Hopkins University Clinical Trials Unit, Byramjee Jeejeebhoy Government Medical College, Pune, Maharashtra, India.
[5] Centre for Tropical Medicine, Nuffield Department of Medicine, University of Oxford, Oxford, UK.
[6] Microbiology Department and Center for Tropical Medicine Research, Ngoc Thach University of Medicine, Ho Chi Minh city, Vietnam.
[7] Yale University, New Haven, CT, USA.
[8] Weill Cornell Medicine, New York, NY, USA.

* Please send all correspondences to: ibrito@cornell.edu.

Supplemental Figures 1-9

Supplemental Figure 1

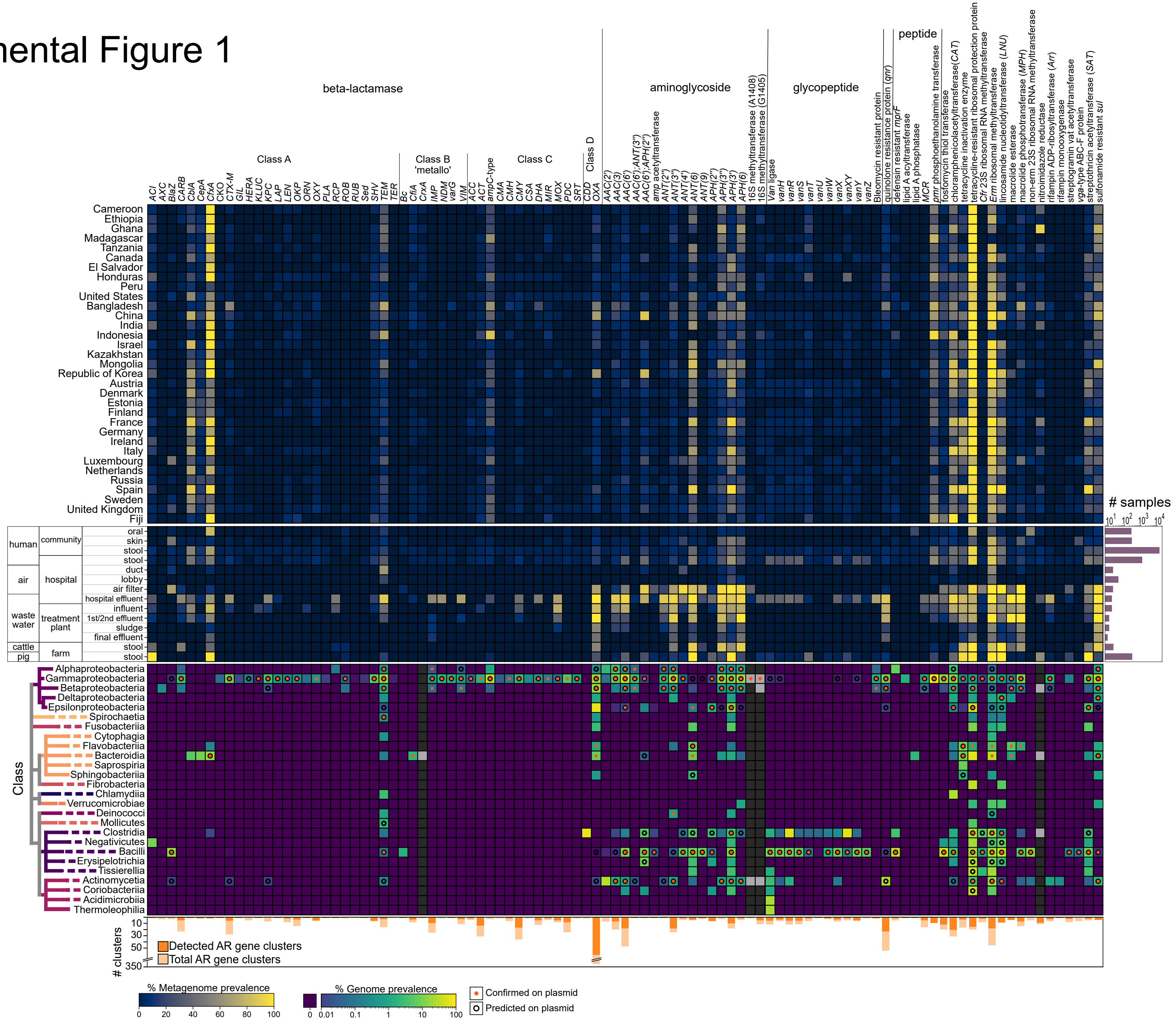

Supplemental Figure 1. Global and taxonomic distribution of AR gene families.
At the top is a heatmap of the prevalence of all AR genes in the CARD protein homolog model, with the exception of efflux pumps and non-housekeeping genes, in human gut-associated microbiomes sequenced from each country. In the middle, AR gene profiles from metagenomes from human- and environmental-associated metagenomes are shown. At the bottom is a heatmap showing the proportions of cultured isolates in each class harboring the AR gene family. At the bottom, a barplot shows the total number of AR gene clusters in each gene family and the number detected in this study in the metagenomic datasets.

Supplemental Figure 2

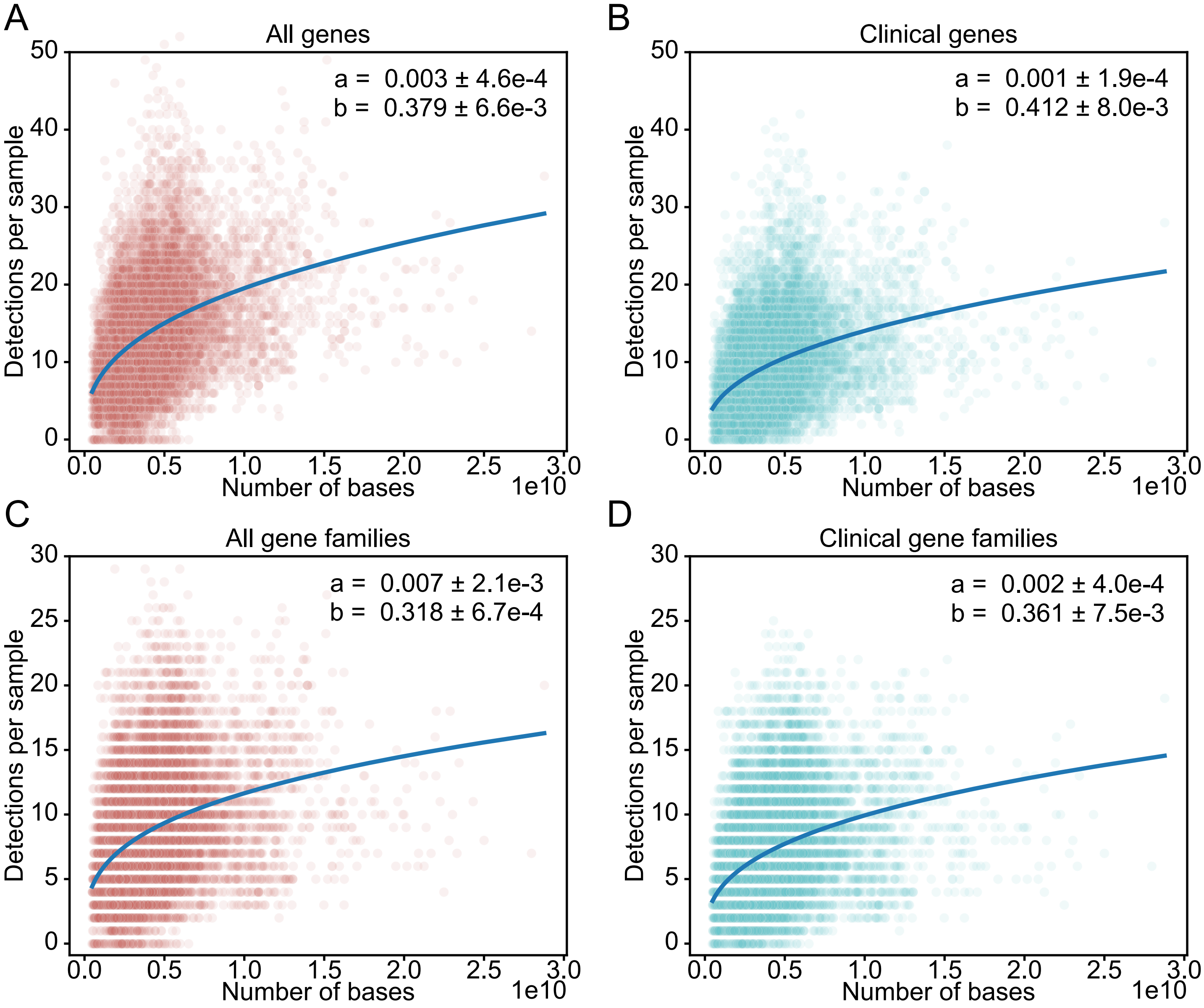

Supplemental Figure 2. Sequencing depth enhances AR gene detection.
The total number of bases is plotted against the total number of AR genes (A) and clinically relevant AR genes (B), AR gene families (C) and clinically relevant AR gene families (D) for each sample. A power regression was used to fit the data.

Supplemental Figure 3

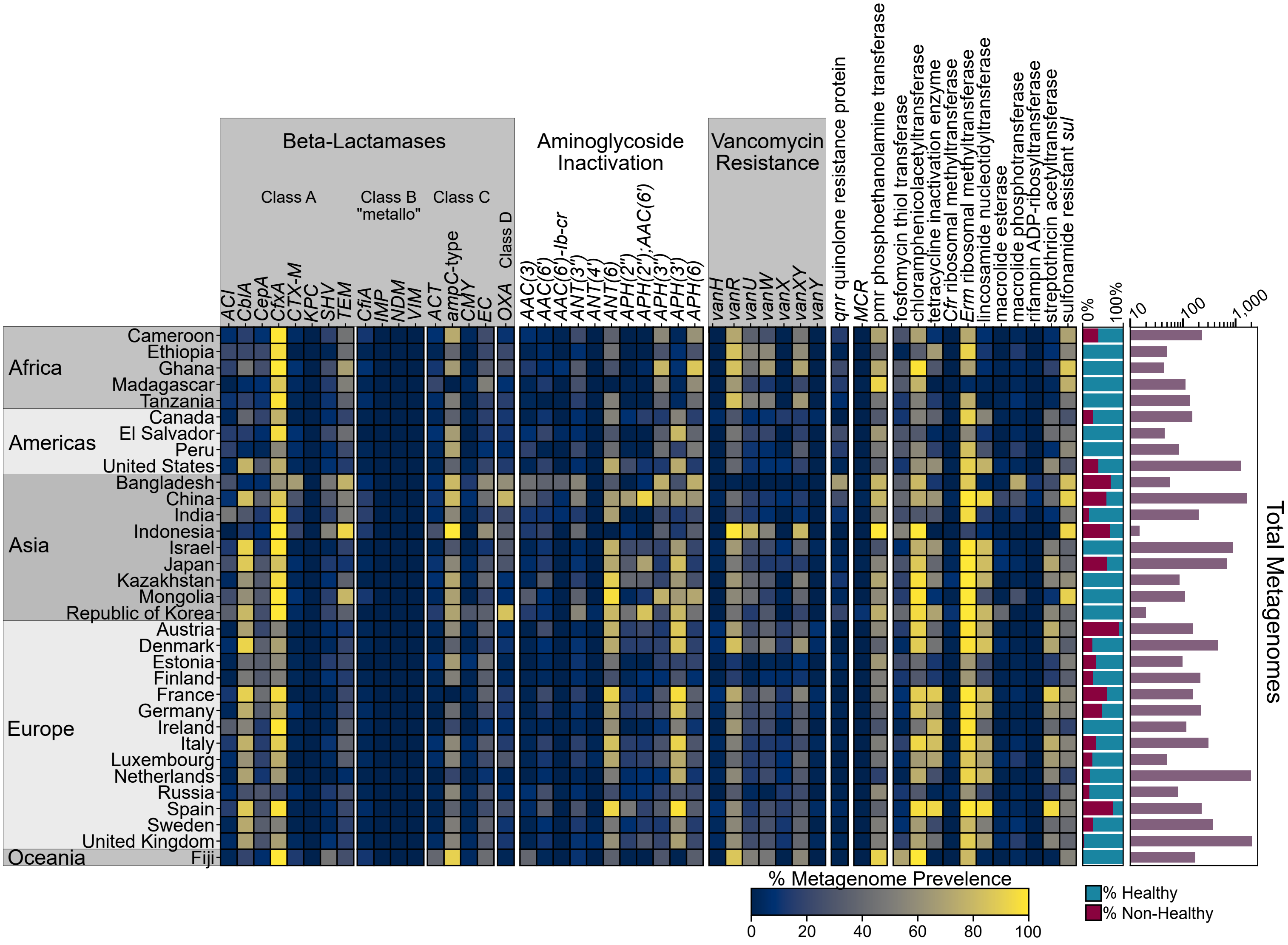

Supplemental Figure 3. Global and taxonomic distribution of AR gene families annotated using UniRef90 proteins in HUMANN.
At the top is a heatmap of the prevalence of all AR genes shown in Figure 1 identified by mapping UniRef90 proteins to AR genes in human gut-associated microbiomes sequenced from each country. At right are two barplots, one showing the proportion of samples from heathy/not-healthy individuals, as classified by curatedMetagenomicDataCuration (left), and a second showing the total number of samples from each country.

Supplemental Figure 4

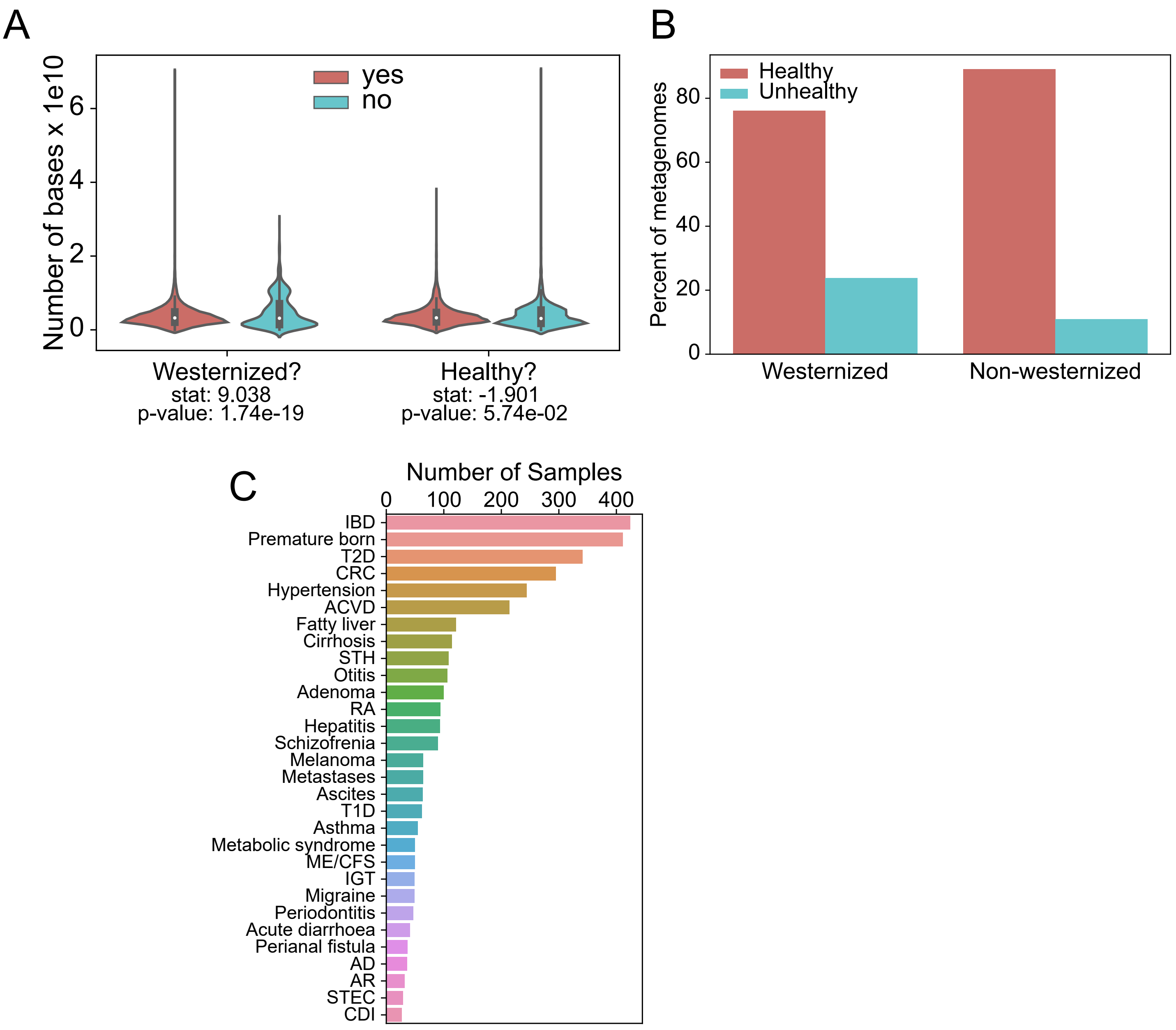

Supplemental Figure 4. Sequencing depth and disease-status for samples from Western and non-Western cohorts.

(A) A violin plot showing the total number of reads per sample for Western and non-Western cohorts (left) and healthy versus non-healthy cohorts (right). (B) A bar plot showing the number of samples from healthy or non-healthy individuals from Western or non-Western cohorts. (C) A chart showing the number of samples derived from non-healthy patients according to disease. Abbreviations: IBD, inflammatory bowel disease; CRC, colorectal cancer; T2D, type 2 diabetes; IGT, impaired glucose tolerance; ACVD, atherosclerotic cardiovascular disease; STH, soil-transmitted helminths; RA, rheumatoid arthritis; T1D, type 1 diabetes; and STEC, Shiga toxin-producing *Escherichia coli* infections.

Supplemental Figure 5

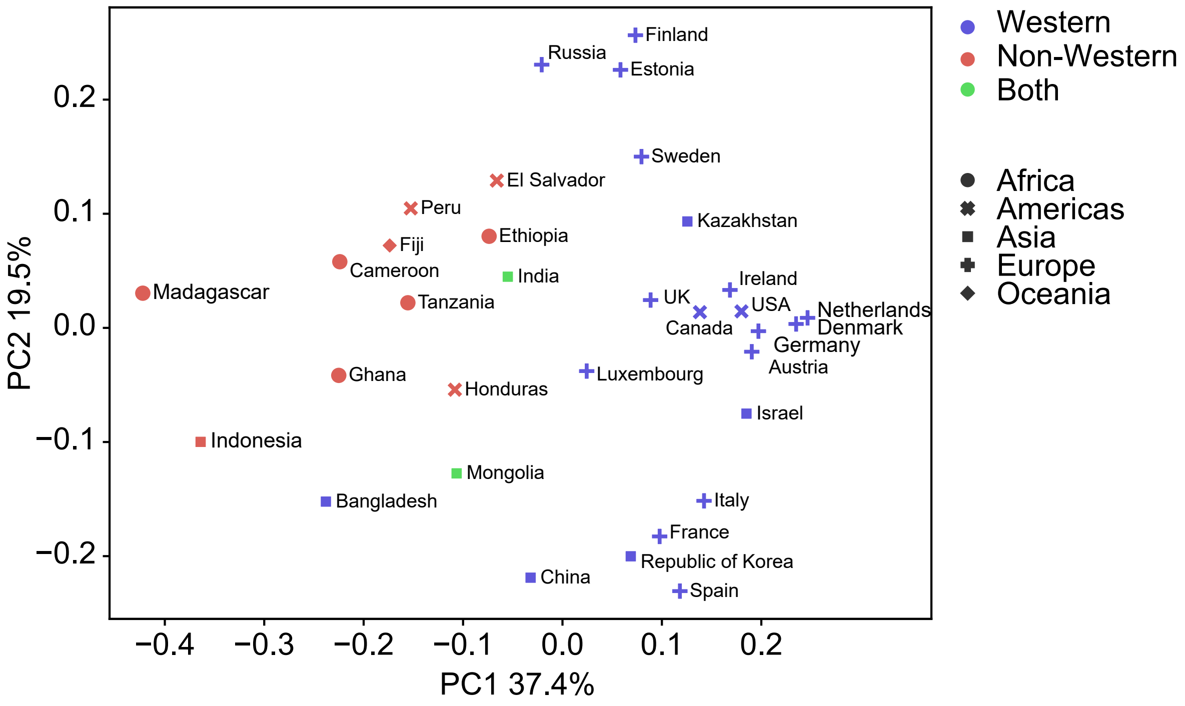

Supplemental Figure 5. PCoA of AR gene resistance profiles per country.
Results of a principal coordinate analysis (PCoA) of AR gene resistance profiles per country showing the first two principal coordinates explaining 56.9% of the variance. Cohorts from each country are colored by Western or non-Western populations.

Supplemental Figure 6

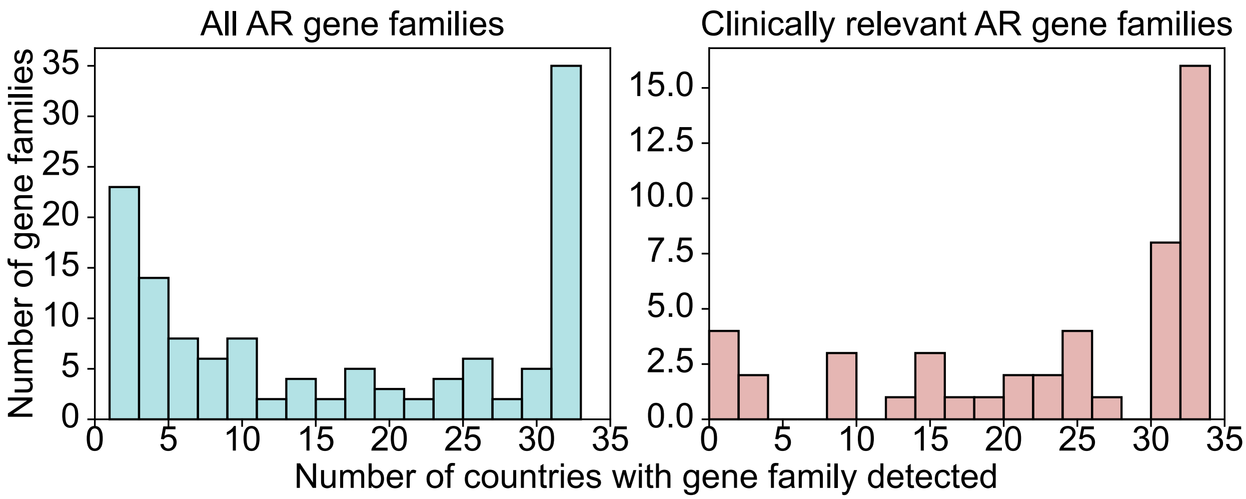

Supplemental Figure 6. AR gene prevalence worldwide is bimodal.
Two histograms showing the number of countries in which each AR gene family (left) or clinically relevant AR gene family (right) was detected.

Supplemental Figure 7

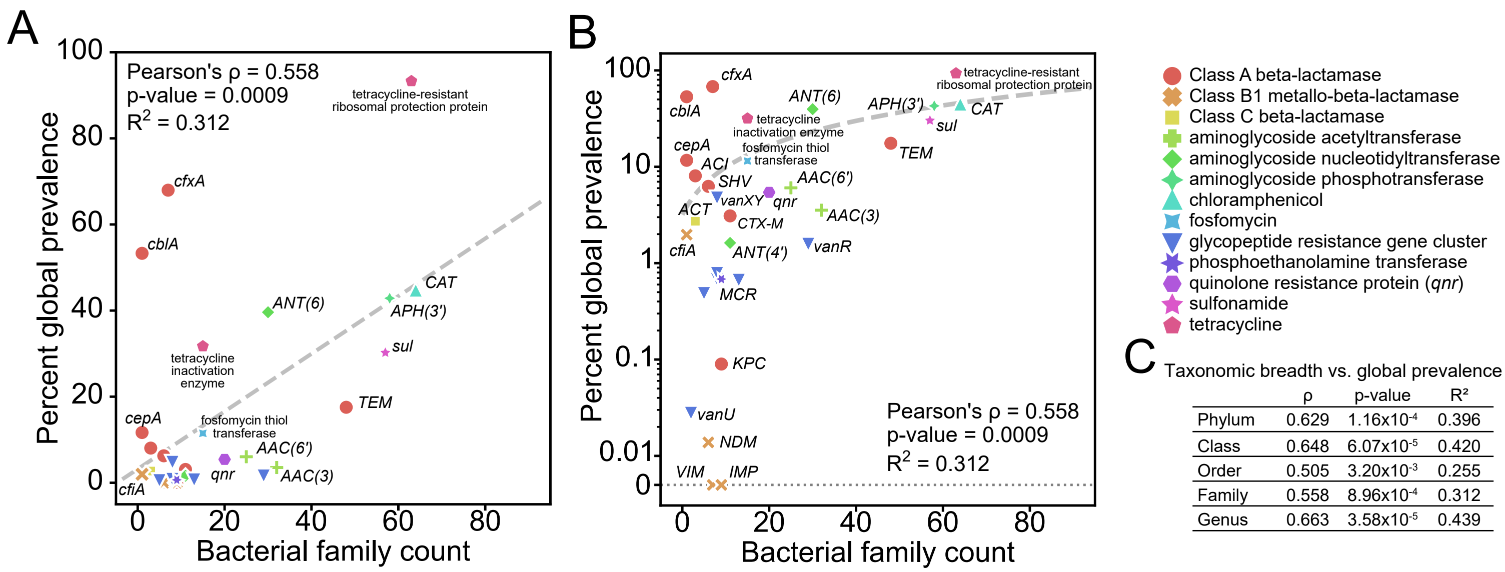

C Taxonomic breadth vs. global prevalence

	ρ	p-value	R^2
Phylum	0.629	1.16×10^{-4}	0.396
Class	0.648	6.07×10^{-5}	0.420
Order	0.505	3.20×10^{-3}	0.255
Family	0.558	8.96×10^{-4}	0.312
Genus	0.663	3.58×10^{-5}	0.439

Supplemental Figure 7. Relationship between taxonomic spread and prevalence of AR gene families.
(A, B) For each AR gene family, the number of bacterial families in which that gene family is found is plotted against the global prevalence of that AR gene family in human microbiomes (a, linear y-axis; b, same data plotted on a logarithmic y-axis). Two-sided Pearson's ρ, p-value and R^2 are reported. (C) Statistics (Two-sided Pearson's ρ, p-value and R^2) are reported for the correlation between taxonomic breadth, at different taxonomic levels, and global prevalence in microbiomes.

Supplemental Figure 8

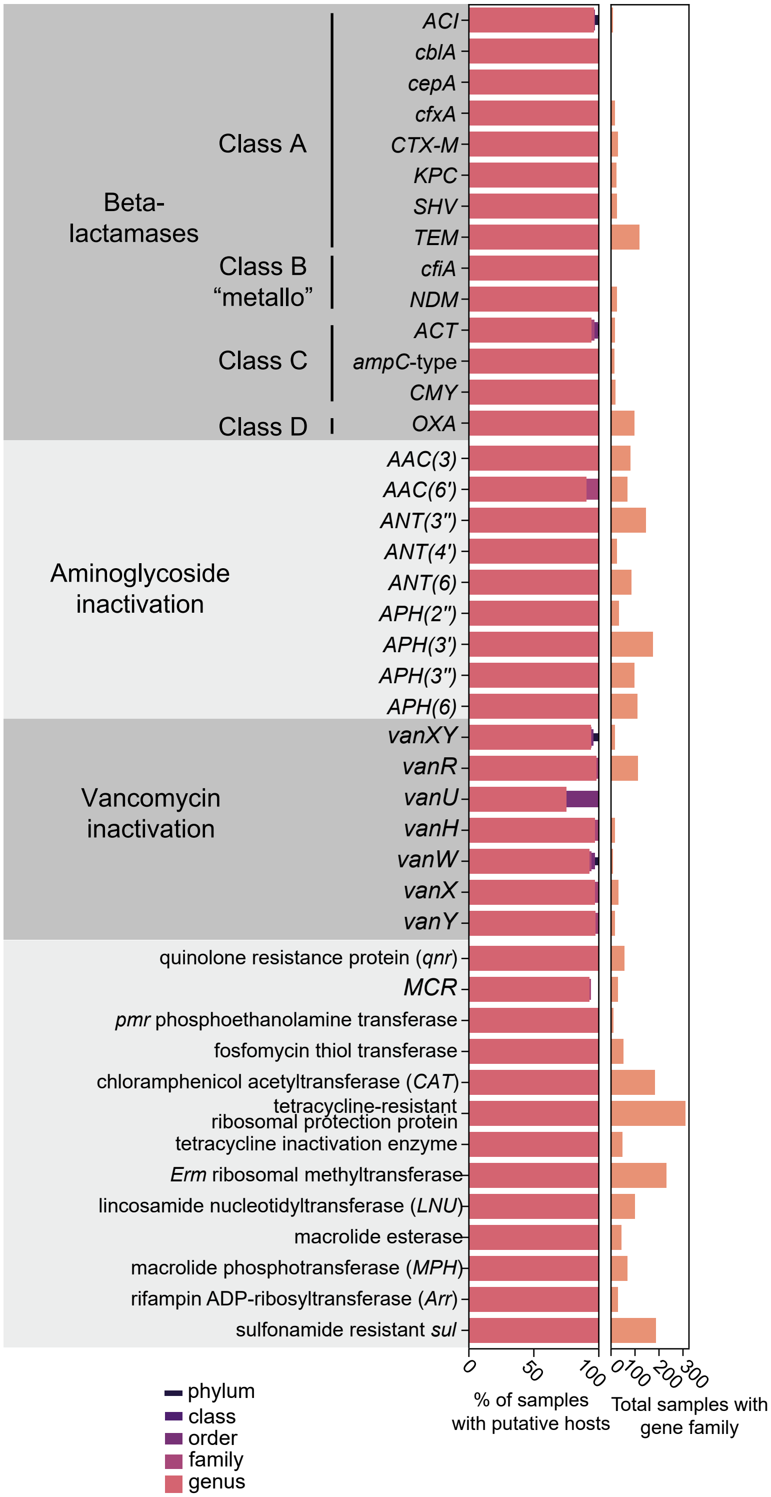

Supplemental Figure 8. AR gene-host associations are sufficiently explained by isolate genomes alone. On the left, for each AR gene family, an overlapping barplot showing the proportion of gut metagenomes that have at least one putative bacterial host, in which an isolate containing the AR gene family has been previously observed. Colors indicate the lowest taxonomic level of potential carriers identified within the metagenome. To the right is the number of total species in which that AR gene family has been observed in isolate genomes.

Supplemental Figure 9

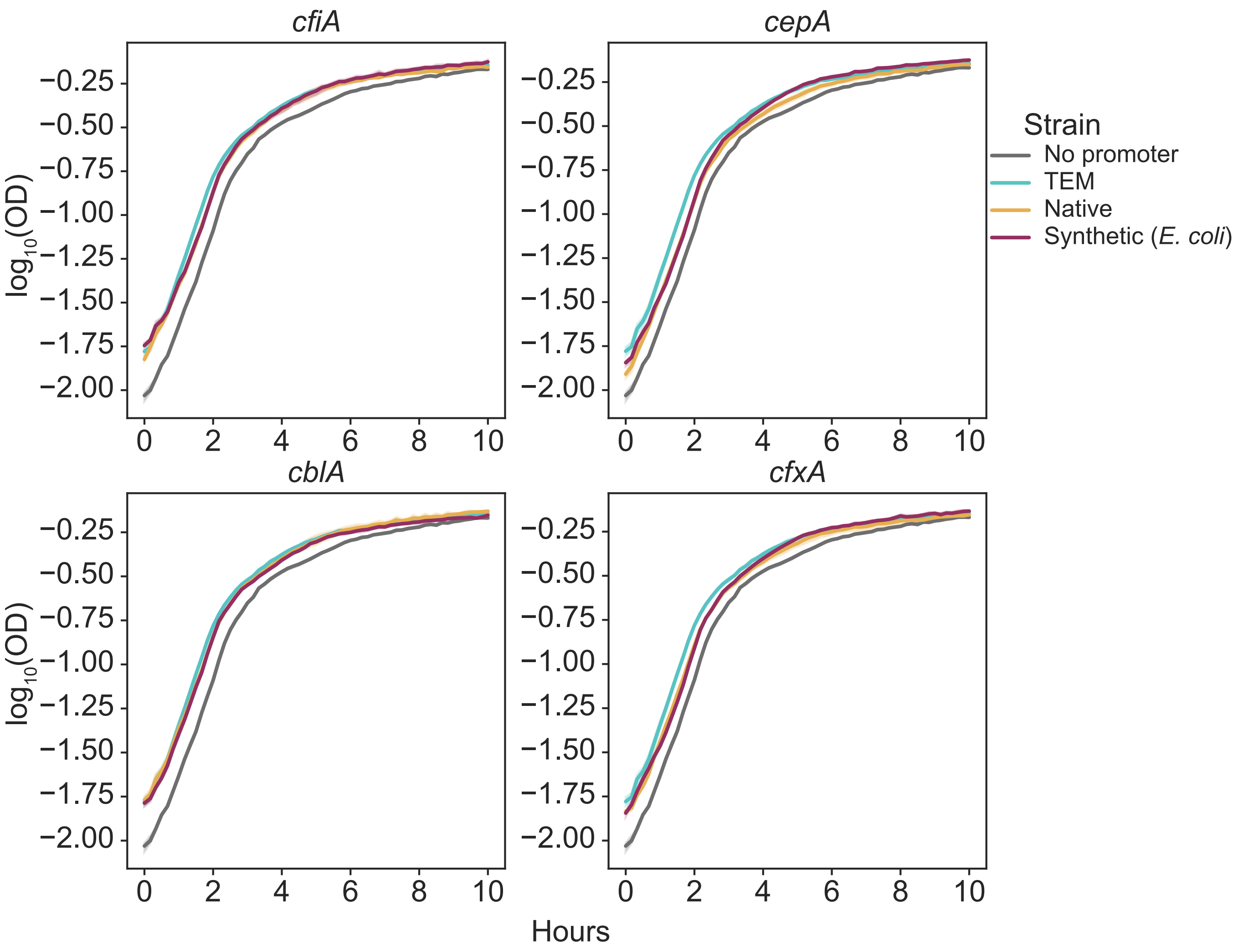

Supplemental Figure 9. Growth curves for *E. coli* harboring various *Bacteroides*-specific beta-lactamase genes.
For each gene listed, the average and standard deviation of three growth curves of *E. coli* with the gene on a plasmid with either no promoter, its native promoter or a synthetic *E. coli* promoter. *E. coli* carrying a comparable plasmid with the *TEM* gene was grown for comparison.
