# Supplementary material for: Clinically relevant antibiotic resistance genes are linked to a limited set of taxa within gut microbiome worldwide
Source: Nat Commun. 2023 Nov 14;14:7366. doi: 10.1038/s41467-023-42998-6 (PMC10645880; doi:10.1038/s41467-023-42998-6)
Supplement: Supplementary file 3 — Descirption of Additional Suppelmentary Data [file 41467_2023_42998_MOESM3_ESM.docx]

**Description of Additional Supplementary Files**

File Name: **Supplementary Data 1**

Description: **Metadata for human metagenomic samples considered in this study.**

A comprehensive table of metadata for the human metagenomes used in Figure 1 and Supplementary Figure 1. The table was adapted from the Waldron lab’s curatedMetagenomicData Bioconductor package25 and contains columns pertaining to sample and participant identifiers, study association with year published, sample type, number of reads, health status, gender, age, and SRA numbers.

File Name: **Supplementary Data 2**

Description: **KMA output of human metagenomes**

Unfiltered KMA output of metagenomes from Supplementary Table 1 aligning trimmed metagenomic reads to the CARD database.

File Name: **Supplementary Data 3**

Description: **AR gene profiles of human metagenomes**

AR gene profiles generated from KMA results in Supplementary Table 2 indicating presence or absence of all AR gene families by sample.

File Name: **Supplementary Data 4**

Description: **Genomes used in this study**

Metadata for all the genomes used in this study, including the taxonomy, ftp path, and file name.

File Name: **Supplementary Data 5**

Description: **RGI results for genomes used in this study**

Unprocessed RGI output from the isolate genomes combined into a single file.

File Name: **Supplementary Data 6**

Description: **List of AR genes included in this study**

A list of AR gene examined as part of this study, along with the class of antibiotics to which they provide resistance and the year of their first report in the scientific literature.

File Name: **Supplementary Data 7**

Description: **Metadata for non-human and inpatient metagenomes**

Curated set of metagenomes from inpatients, hospital air and wastewater, wastewater treatment plants, and farm animals

File Name: **Supplementary Data 8**

Description: **KMA output from non-human and inpatient metagenomes**

Unfiltered KMA output of metagenomes from Supplementary Table 7 aligning trimmed metagenomic reads to the CARD database

File Name: **Supplementary Data 9**

Description: **AR gene profiles from non-human and inpatient metagenomes**

AR gene profiles generated from KMA results in Supplementary Table 8 indicating presence or absence of all AR gene families by sample.

File Name: **Supplementary Data 10**

Description: **List of primers used for OIL-PCR, qPCR, and cloning**

File Name: **Supplementary Data 11**

Description: **List of OIL-PCR and 16S sequencing libraries**

List of sequencing libraries used to generate Figure 3.
