# Supplementary material for: Clinically relevant antibiotic resistance genes are linked to a limited set of taxa within gut microbiome worldwide
Source: Nat Commun. 2023 Nov 14;14:7366. doi: 10.1038/s41467-023-42998-6 (PMC10645880; doi:10.1038/s41467-023-42998-6)
Supplement: Supplementary file 15 — Reporting Summary [file 41467_2023_42998_MOESM15_ESM.pdf]

nature portfolio

Corresponding author(s): Peter Diebold, Matthew Rhee, Qiaojuan Shi, Nguyen Vinh Trung, Ngo Thi Hoa, Nicholas Christakis, Najeeha Iqbal, Asad Ali, Jyoti Mathad, Ilana Lauren Brito

Last updated by author(s): 10-22-23

Reporting Summary

Nature Portfolio wishes to improve the reproducibility of the work that we publish. This form provides structure for consistency and transparency in reporting. For further information on Nature Portfolio policies, see our Editorial Policies and the Editorial Policy Checklist.

Statistics

For all statistical analyses, confirm that the following items are present in the figure legend, table legend, main text, or Methods section.

n/a	Confirmed	
☐	☒	The exact sample size (n) for each experimental group/condition, given as a discrete number and unit of measurement
☐	☒	A statement on whether measurements were taken from distinct samples or whether the same sample was measured repeatedly
☐	☒	The statistical test(s) used AND whether they are one- or two-sided<br>*Only common tests should be described solely by name; describe more complex techniques in the Methods section.*
☒	☐	A description of all covariates tested
☒	☐	A description of any assumptions or corrections, such as tests of normality and adjustment for multiple comparisons
☐	☒	A full description of the statistical parameters including central tendency (e.g. means) or other basic estimates (e.g. regression coefficient) AND variation (e.g. standard deviation) or associated estimates of uncertainty (e.g. confidence intervals)
☐	☒	For null hypothesis testing, the test statistic (e.g. F, t, r) with confidence intervals, effect sizes, degrees of freedom and P value noted<br>*Give P values as exact values whenever suitable.*
☒	☐	For Bayesian analysis, information on the choice of priors and Markov chain Monte Carlo settings
☒	☐	For hierarchical and complex designs, identification of the appropriate level for tests and full reporting of outcomes
☐	☒	Estimates of effect sizes (e.g. Cohen's d, Pearson's r), indicating how they were calculated

Our web collection on statistics for biologists contains articles on many of the points above.

Software and code

Policy information about availability of computer code

Data collection	Genomes assemblies were downloaded via FTP from NCBI and metagenomic reads were downloaded via FTP from EBI.
Data analysis	bbTools version 38.96, KMA version 1.4.3 RGI version 5.2.0 , PlasForest version1.4, usearch version 11.0.667 , CD-HIT, Cutadapt version 3.4

For manuscripts utilizing custom algorithms or software that are central to the research but not yet described in published literature, software must be made available to editors and reviewers. We strongly encourage code deposition in a community repository (e.g. GitHub). See the Nature Portfolio guidelines for submitting code & software for further information.

Data

Policy information about availability of data

All manuscripts must include a data availability statement. This statement should provide the following information, where applicable:

- Accession codes, unique identifiers, or web links for publicly available datasets
- A description of any restrictions on data availability
- For clinical datasets or third party data, please ensure that the statement adheres to our policy

Genomes were downloaded with FTP from genbank and plasmid sequences from refseq/plasmids. FTP links for metagenomic reads were compiled from the 'curatedMetagenomicData" package and downloaded from EBI. Additional metagenomes were curated from the literature and downloaded in the same manner.

CARD database version 3.1.4

Metadata for genomes, plasmids, and metagenomes are available in tables 1, 4, and 7. These tables include accession umbers and FTP links. Sequencing is uploaded to SRA (PRJNA999635, PRJNA999651,PRJNA1001934)

Human research participants

Policy information about studies involving human research participants and Sex and Gender in Research.

Reporting on sex and gender	Vietname, Pakistan, and Honduraas samples included both ment and women. India samples were only women.
Population characteristics	Indian samples were all pregnant women.
Recruitment	Honduran study participants from 9 isolated villages in the western highlands of Honduras that were part of larger population-based cohort assembled for a different purpose97 were asked to take part in this study. The goal of the microbiome sampling was to be as comprehensive as possible. Pakistani study participants comprised adults (over the age of 18) recruited via the existing community-based antimicrobial surveillance system established by the two union councils of the Matiari district. Participants were stratified based on ethnicity/caste and tribe and random representatives were chose across the communities. Vietnamese participants comprised adult farmers (over the age of 18) involved in studies conducted by the Oxford University Clinical Research Unit (OUCRU) in Vietnam. Indian participants comprised a subset of pregnant women enrolled in the PRACHITi study in Pune, India98. All women were over the age of 18 who presented to the antenatal clinic at BJ Government Medical College in Pune, India, with gestational age between 13–34 weeks.
Ethics oversight	Human subjects research was approved by the following committees: Cornell University Institutional Review Board (#1706007261, # 1702006922), Aga Khan University Institutional Review Board (# 2018-0550-513), Ethics Committee of the University of Oxford (OxTREC 38-15) and of Tien Giang Hospital Institutional Review Board (278/BVĐK), the Yale University Institutional Review Board (#2000020688), the BJ Government Medical College Ethics Board and Weill Cornell Medicine Institutional Review Board (#1503016041)

Note that full information on the approval of the study protocol must also be provided in the manuscript.

Field-specific reporting

Please select the one below that is the best fit for your research. If you are not sure, read the appropriate sections before making your selection.

☒ Life sciences ☐ Behavioural & social sciences ☐ Ecological, evolutionary & environmental sciences

For a reference copy of the document with all sections, see nature.com/documents/nr-reporting-summary-flat.pdf

Life sciences study design

All studies must disclose on these points even when the disclosure is negative.

Sample size	Sample size for profiling ARG family prevalence globally and taxonomically was determined by the number of available genomes and metagenomes. Sample size for OIL-PCR was based on the number of samples which could be processed.
Data exclusions	Some samples did not amplify well with OIL-PCR, or detections were not seen across replicates. These samples were excluded from further analysis
Replication	The global metagenomic analysis was performed using KMA on raw reads as well as HUMAnN3 results from the curatedMetagenomicData package. Results strongly mirrored each other.<br><br>qPCR and OIL-PCR experiments were performed in triplicate. Growth curves and promoter sequencing was also performed in triplicate.
Randomization	This study was a targeted screen for specific AR genes in stool. We performed an initial pre-screen to identify samples which carried the genes of interest. In this design, randomization would not be informative because we had already selected samples based on this criteria.
Blinding	Similar to randomization, the samples were chosen for a specific criteria. All analysis was performed in parallel computationally and would not be biased by the data scientist.

Reporting for specific materials, systems and methods

We require information from authors about some types of materials, experimental systems and methods used in many studies. Here, indicate whether each material, system or method listed is relevant to your study. If you are not sure if a list item applies to your research, read the appropriate section before selecting a response.

Materials & experimental systems

n/a	Involved in the study
☒	☐ Antibodies
☒	☐ Eukaryotic cell lines
☒	☐ Palaeontology and archaeology
☒	☐ Animals and other organisms
☒	☐ Clinical data
☒	☐ Dual use research of concern

Methods

n/a	Involved in the study
☒	☐ ChIP-seq
☒	☐ Flow cytometry
☒	☐ MRI-based neuroimaging
