# Supplementary material for: Clinically relevant antibiotic resistance genes are linked to a limited set of taxa within gut microbiome worldwide
Source: Nat Commun. 2023 Nov 14;14:7366. doi: 10.1038/s41467-023-42998-6 (PMC10645880; doi:10.1038/s41467-023-42998-6)
Supplement: Supplementary file 2 — Peer Review File [file 41467_2023_42998_MOESM2_ESM.pdf]

nature portfolio

Peer Review File

Clinically relevant antibiotic resistance genes are linked to a limited set of taxa within gut microbiome worldwide

Open Access This file is licensed under a Creative Commons Attribution 4.0 International License, which permits use, sharing, adaptation, distribution and reproduction in any medium or format, as long as you give appropriate credit to the original author(s) and the source, provide a link to the Creative Commons license, and indicate if changes were made. In the cases where the authors are anonymous, such as is the case for the reports of anonymous peer reviewers, author attribution should be to 'Anonymous Referee' followed by a clear attribution to the source work. The images or other third party material in this file are included in the article's Creative Commons license, unless indicated otherwise in a credit line to the material. If material is not included in the article's Creative Commons license and your intended use is not permitted by statutory regulation or exceeds the permitted use, you will need to obtain permission directly from the copyright holder. To view a copy of this license, visit http://creativecommons.org/licenses/by/4.0/.

Editorial Note: This manuscript has been previously reviewed at another journal that is not operating a transparent peer review scheme. This document only contains reviewer comments and rebuttal letters for versions considered at *Nature Communications.*

REVIEWERS' COMMENTS

Reviewer #1 (Remarks to the Author):

I think the authors have done a nice job adressing the reviews.

Nothing further to add.

Reviewer #2 (Remarks to the Author):

The authors have adequately addressed my concerns- I have no remaining comments (and reading response to the other reviewers, feel the authors did a great job addressing all the criticisms overall)

Reviewer #3 (Remarks to the Author):

The reviewer appreciates the authors' additional analyses and revisions to the manuscript. The conclusions remain interesting, and the revisions strengthen the work. The reviewer does not have any other concerns.
